# Opioid-Sparing Options in Perioperative Pain Management: An Expert Video Roundtable Discussion

**DOI:** 10.1093/asjof/ojag153

**Published:** 2026-08-04

**Authors:** Al Aly, Rodney J Rohrich, Nicholas Haddock, Sam Jejurikar

The authors are pleased to present an expert roundtable discussion on nonopioid pain management in aesthetic plastic surgery (Video). The discussants include a panel of leading experts in aesthetic surgery, and the video roundtable was recorded live in Dallas, TX. The discussion began with an overview of Journavx (Vertex Pharmaceuticals, Boston, MA), the first FDA-approved nonopioid oral analgesic for postoperative pain. The drug is a sodium channel blocker with similar efficacy to opioids and few side effects. The panelists agreed that it has a wide range of uses in plastic surgery across a variety of procedures, from body contouring surgery and breast reconstruction to less invasive procedures. Although all panelists agreed that the drug has become an important aspect of their pain management protocols, they have seen issues with inconsistent availability and high costs for patients, because of the newness of the drug. Despite these current issues, the panelists remain excited about the potential for this drug to become an important aspect of pain management in aesthetic plastic surgery and beyond.

## Supplemental Material

This article contains supplemental material located online at www.asjopenforum.com.

## Supplementary Material

ojag153_Supplementary_Data

